# Three-Dimensional Reconstructions Come to Life – Interactive 3D PDF Animations in Functional Morphology

**DOI:** 10.1371/journal.pone.0102355

**Published:** 2014-07-16

**Authors:** Thomas van de Kamp, Tomy dos Santos Rolo, Patrik Vagovič, Tilo Baumbach, Alexander Riedel

**Affiliations:** 1 ANKA/Institute for Photon Science and Synchrotron Radiation, Karlsruhe Institute of Technology (KIT), Eggenstein-Leopoldshafen, Germany; 2 State Museum of Natural History (SMNK), Karlsruhe, Germany; Monash University, Australia

## Abstract

Digital surface mesh models based on segmented datasets have become an integral part of studies on animal anatomy and functional morphology; usually, they are published as static images, movies or as interactive PDF files. We demonstrate the use of animated 3D models embedded in PDF documents, which combine the advantages of both movie and interactivity, based on the example of preserved *Trigonopterus* weevils. The method is particularly suitable to simulate joints with largely deterministic movements due to precise form closure. We illustrate the function of an individual screw-and-nut type hip joint and proceed to the complex movements of the entire insect attaining a defence position. This posture is achieved by a specific cascade of movements: Head and legs interlock mutually and with specific features of thorax and the first abdominal ventrite, presumably to increase the mechanical stability of the beetle and to maintain the defence position with minimal muscle activity. The deterministic interaction of accurately fitting body parts follows a defined sequence, which resembles a piece of engineering.

## Introduction

Functional morphology of animals usually relies on observations of living specimens and/or the interpretation of morphological characters found in dead ones [1]. In recent years, the arrival of three-dimensional (3D) imaging techniques significantly extended the pool of available methods for morphological studies [2]–[5]. Digital models based on segmented datasets allow the analysis of both external and internal structures [6], and by providing a “digital copy” they facilitate a non-destructive examination of minute, brittle, and irreplaceable samples. Some animations of 3D data have been published recently as 2D movies [7]–[11].

Animated 3D PDF (portable document format) files, however, provide a much broader range of interactivity as opposed to movies, as the perspective can be chosen and varied and/or complex models can be masked to show only selected parts of interest, e.g. distinct muscle groups or parts of the skeleton [12], [13]. Most software applications used for image stack segmentation do not offer sufficient functionality to move polygon meshes with respect to each other. Herein, we describe an approach to analyse and illustrate complex motion systems by animating 3D mesh models of static specimens with the help of 3D animation software.

**Figure 1:**
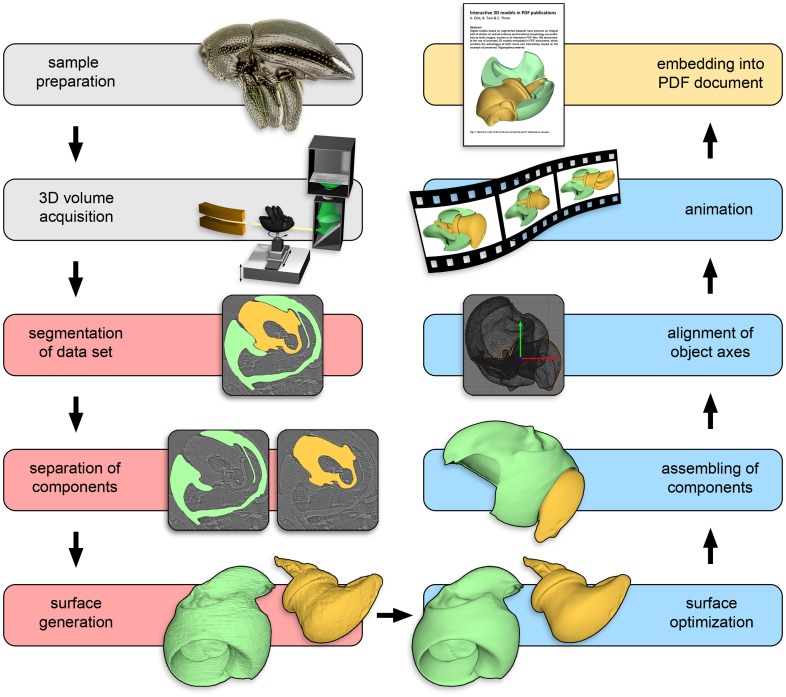
Flow diagram of the steps creating an interactive animated 3D model, based on the example of a screw-and-nut type hip joint of the weevil *Trigonopterus oblongus*. After acquisition of a 3D volume, scientific visualization software (e.g. Amira; red boxes) is used for creating surface models. 3D computer graphics software (here: CINEMA 4D and Deep Exploration; blue boxes) is employed for surface optimization, assembling and animation. The animated model may be embedded into a PDF document.

We illustrate the workflow (Figure 1) based on μCT (synchrotron X-ray microtomography) data of *Trigonopterus* weevils [14]: First, the hind leg's screw-and-nut type joint [15] is animated (Figure S1); we proceed with the animation of the entire weevil, i.e., a motion system comprising 44 components (Figure S2), to clarify the functional morphology of its defensive behaviour. The latter involves death-feigning, also known as thanatosis [16]. When preparing preserved specimens we found it hard to move their rostrum and legs from thanatosis into a walking position. Movements appeared mechanically blocked and it was impossible to identify the blocking mechanism by manual examination.

*Trigonopterus* Fauvel is a genus of wingless weevils dwelling in primary forests of Southeast Asia and Melanesia. Its hundreds of species are spread over its range, many of them still undescribed. New Guinea appears to be a centre of its diversity with more than 300 species recorded [17], [18]. Specimens are found sitting on foliage or in the litter of forest floors, but little is known of their biology. A compact thanatosis position may be a character that gained evolutionary significance with *Trigonopterus*' inability to fly. Thus, a full understanding of the passive defence mechanisms may lead to a better understanding of the genus' extraordinary diversity.

## Materials and Methods

### Samples

We scanned two complete specimens of *Trigonopterus vandekampi* Riedel [19] of similar body size and one specimen of *Trigonopterus oblongus* (Pascoe). One specimen of *T. vandekampi* was in walking, the others in thanatosis position. All specimens had been fixed in 100% ethanol and were critical point dried.

### Synchrotron-based X-ray microtomography

Tomographic scans were performed at the microtomographic station at the TOPO-TOMO beamline of the ANKA synchrotron radiation facility located at Karlsruhe Institute of Technology, Germany. The tomographic 180° scans were taken using a filtered white beam with the spectrum peak at about 20 keV. An indirect detector system based on scintillating screen, diffraction limited optical microscope and CCD detector was used for the acquisition of the frames. For converting X-rays into visible light an LSO terbium doped scintillator was employed.

In the case of *T. oblongus*, a magnification of 18× resulted in an effective pixel size of 0.5 µm. An exposure time of 240 ms per frame was used to record 2,500 projections. Both specimens of *T. vandekampi* were scanned with a magnification of 10× and an effective pixel size of 0.9 µm. An exposure time of 2 s per frame was used to record 1,500 projections. For all scans, a PCO 4000 14 bit CCD camera system with a resolution of 4,008×2,672 pixels served for recording the frames. Before reconstruction, the frames were processed with the phase retrieval ImageJ plugin ANKAphase [20]. Volume reconstruction was done with the PyHST software developed at the European Synchrotron Radiation Facility in Grenoble, France. Microtomographic image data are deposited in MorphDBase (accession numbers A_Riedel_20140623-M-10.1, A_Riedel_20140623-M-19.1, A_Riedel_20140623-M-15.1, A_Riedel_20140623-M-13.1, A_Riedel_20140623-M-16.1, A_Riedel_20140623-M-17.1)

### Segmentation

Body sclerites were segmented and converted into individual surface components (polygon meshes), as done in other recent studies [21]–[23] following the procedure described in [12]. Soft tissue and connecting cuticle were not segmented unless hard to delimit from sclerites, i.e. at the attachment points of tendons. The 3D volumes were imported into Amira (version 5.4.2; FEI Visualization Sciences Group) or Avizo (version 6.2.1; FEI Visualization Sciences Group). The Image slices were segmented manually to create polygon meshes (surface models). Initially, every tenth slice was segmented with subsequent interpolation on interjacent slices. For delicate structures, smaller steps were taken to minimize interpolation errors. The interpolated labels were checked; errors and artefacts were corrected manually. After segmenting the objects each morphological structure was isolated. The *smooth labels* dialog was used for smoothing the labels (size 5; mode: 3D volume) and polygon meshes of the structures' surfaces were created with the *SurfaceGen* module at default settings.

### Optimization of polygon meshes

Polygon meshes from segmented image volumes typically contain millions of polygons and numerous segmentation artefacts showing the traces of individual layers. A smooth surface facilitates reduction of the polygon count without losing too many structural details. Thus, an iterative series of surface smoothing and polygon reduction is most effective in removing segmentation artefacts and simultaneously reducing polygon count to 0.1% (Figure S3) thus greatly helping data handling in the downstream process.

For this study, the polygon count of the original meshes was reduced to 10% in Amira/Avizo. The files were subsequently saved in the Wavefront format (OBJ) to allow import into CINEMA 4D (versions 12 & 14; Maxon Computer GmbH) for subsequent smoothing and polygon reduction. The parameters were set with respect to the polygon count and the general shape of the objects.

### Axis alignment, motion analysis and animation

Surface meshes may be animated using any suitable 3D program from a wide choice of software. For embedding an animated model into a 3D PDF document, the data have to be saved as Universal 3D Files (U3D) using e.g. Deep Exploration. Here, we used CINEMA 4D (Version 14) in the case of *T. oblongus* and Deep Exploration (Version 6; Right Hemisphere®; Note S2) to animate the joints of *T. vandekampi*.

Before animation, all meshes were assembled in CINEMA 4D with each component separately editable. Based on the position of the segmented sclerites in the original image stack, the individual components are automatically placed at their correct positions in the software's coordinate system. For the complex model of *T. vandekampi*, symmetric appendices (i.e. antennae and legs) were duplicated and mirrored. Object hierarchies were created and meshes of the different body parts were coloured.

**Figure 2:**
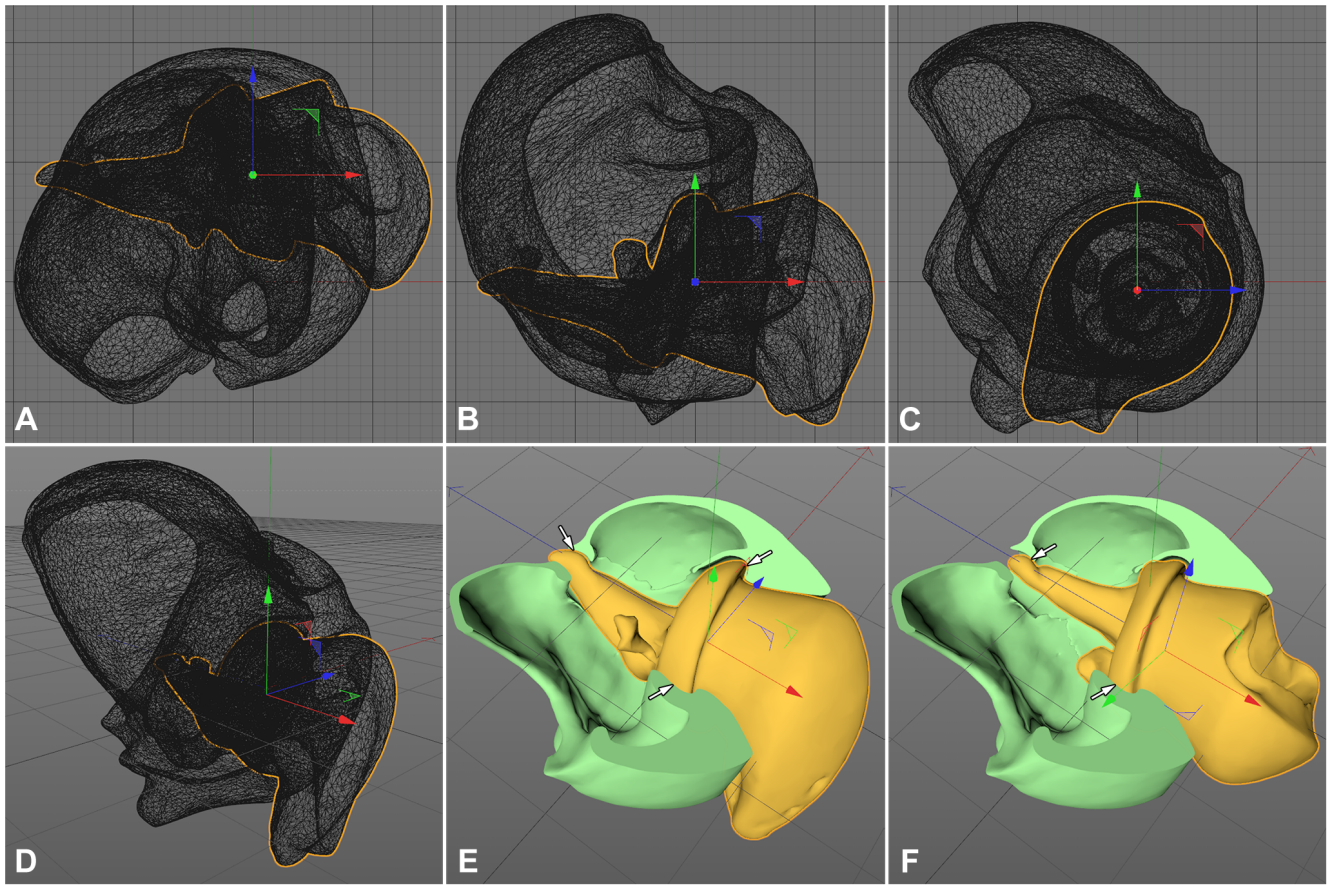
Axis alignment and animation of the screw joint of *Trigonopterus oblongus* in CINEMA 4D. (A–C) 2D views (A: bottom, B: right, C: front) displaying surface isoparms for axis alignment. The boundary of the trochanter is indicated by the yellow frame, the rotation axis by the arrows (red: X axis, green: Y axis, blue: Z axis). (**D**) Displayed surface isoparms in central perspective. (**E, F**) Same joint (Gouraud shading); coxa (green) cut by attached Boole tool, thus revealing friction surfaces of the joint parts (white arrows).

Most joints of the heavily sclerotized weevil show a precise form closure of its components, so possible movements could be simulated by interactively moving one component towards its counterpart until the joint reaches the fully bent, respectively depressed position, yet avoiding any overlap of the adjacent surfaces. The joint's motion could be approximated by iterative trial and error. First, an appropriate position for the animation axes had to be found for each component of the joint. The axes were aligned by using the software's object axis tool (Figure 2). The position of an object axis was altered from three 2D perspectives (bottom, right and front view) to determine the optimal position in three-dimensional space. Positioning of the axis is highly sensitive and a tilting of only 0.1° from the ideal position may visibly increase artificial overlap of surfaces.

Then, one component was moved relative to the other finding its terminal positions, i.e. its fully extended and its fully depressed position, and for each a keyframe was created, thus defining the beginning and the end of the motion. Intermediate frames were interpolated automatically using linear interpolation setting. In joints with simple movements, e.g. a rotation around one stable axis, these two terminal keyframes were enough to simulate the joint's motion satisfactorily. However, in most cases the position of an animated component required realignment during the movement, and between two and six additional keyframes at intermediate positions had to ensure a precise simulation. During this process of approximating an optimal simulation, invalid arrangements could be detected by overlapping surfaces with display settings to isoparms in different 2D perspectives (e.g. bottom, right, front (Figure 2 A–C). In addition, the joints were temporarily cut to reveal any unrealistic friction of surfaces (Figure 2 E,F). Hard, guiding surfaces and soft structures, e.g. membranes or flexible tendons, which are pushed aside during movement in the living animal, had to be distinguished by the investigator.

The specimen with extended legs was segmented in part to verify the terminal position of the metacoxa. Its cavity is anteriorly open, so its movement is not strictly confined by the thorax (as is the case in pro- and mesocoxa), and thus required empiric measurement of its position with legs extended. From both positions, groups of polygon models composed of the metacoxa, metatrochanter, metafemur and parts of thorax and abdomen were loaded into the same scene and scaled to the same size. The walking position group was moved until thorax and abdomen overlapped with the ones from thanatosis. Thus assigning the final positions of the hind leg, we simulated its movement from walking position to thanatosis. Based on our field observations, the whole process of attaining thanatosis position in *Trigonopterus* takes about one second, i.e. it is faster than the eye can follow in detail. Thus, we decreased the motion speed of our animation. The precise timing of each joint's motion is considered a working hypothesis, since no video recording of the process is available. The adduction of all joints starts simultaneously as is the case in many other weevils falling into thanatosis.

Between 120 and 180 frames for the animation of each joint allowed smooth interpolation and an overall animation time of several seconds at 30 fps (Figure S2). The model of the screw joint of *T. oblongus*, which was animated in CINEMA 4D, was saved as a COLLADA 1.4 file (DAE). and imported into Deep Exploration.

For both models Deep Exploration was used to colour the mesh components and to create the final hierarchies for the meshes. Animation speed was set to 30 fps. Each model including materials and animations was subsequently saved as a Universal 3D File (U3D), containing both mesh geometry and animation sequences.It can be opened and displayed with suitable software, e.g. Deep Exploration, but for a wide dissemination the PDF format is preferable.

### Embedding into PDF files

New documents were created with Adobe Acrobat (version 9 Pro Extended; Note S2) and the U3D meshes were implemented with the *3D tool*. Using default *Activation Settings* and assigning a *Poster Image* from default view, the 3D visualization parameters were set as follows: white background, CAD optimized lights, solid rendering style and default 3D conversion settings. For the reconstruction of the coxa-trochanteral joints of *T. oblongus*, the animation style was set to *Bounce*, whereas it was set to *Loop* for the animated reconstruction of *T. vandekampi*. After starting the 3D view by clicking on the poster image, several views were created using the *Manage Views* option from the 3D toolbar. Annotations were added to the documents, which were subsequently saved as Portable Document Format files (PDF). Animated models are deposited at Dryad (http://doi.org/10.5061/dryad.56kf4).

## Results

### Animation of a screw joint

For the isolated metacoxal screw joint, each coxa and trochanter were segmented separately (Figure S3). The terminal keyframes were set at 0 and 120, and four additional keyframes were needed to ensure realistic simulation for an arbitrary animation time of four seconds. The animation shows a rotation of 130 degrees with a translatory movement of 65 µm. Besides its larger size, the metacoxal joint of *T. oblongus* appears similar or identical to that of *T. vandekampi*.

### Animation of a complex system - thanatosis of a Trigonopterus weevil

A digital model of *T. vandekampi* suitable to answer our questions pertaining to the functional morphology of thanatosis was created by segmenting the major body sclerites (Table S1) of the specimen in thanatosis and by animating 50 individual articulations (Table S2). The noteautomatic placement of the individual components (i.e. the corresponding joint partners) in a consistent coordinate system as assigned by the software Amira resulted in an accurate animation of the assembled virtual beetle (Figure S2).

### Trigonopterus weevil's cascade of movements to attain thanatosis

**Figure 3:**
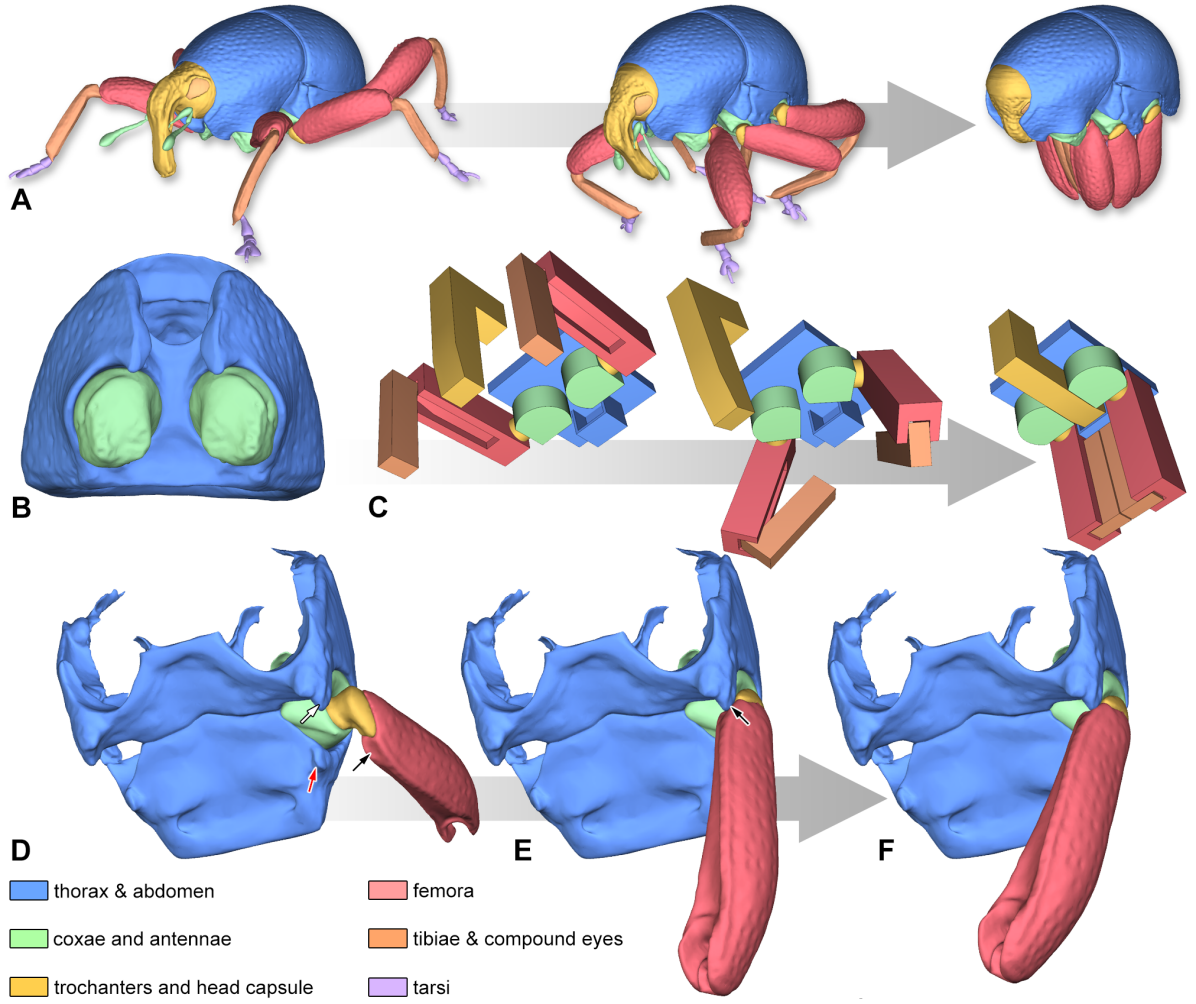
Blocking mechanisms of legs in *Trigonopterus vandekampi*. (A) Illustration of the movement from walking position to thanatosis. (**B**) Prothorax in ventral aspect; note the flattened mesial faces of the coxae and the narrow thoracic canal. (**C**) Simplified model of the prothoracic blocking mechanism. (**D–F**) Metacoxal leverage. (**D**) Hind leg elevated; note the depressed face of the metafemur (black arrow), the metathoracic intercoxal ridge (white arrow) and the abdominal protrusion (red arrow). (**E**) Inward rotation of the trochanter causes the depressed face of the femur to press against the posterior face of the intercoxal ridge (arrow). (**F**) The leverage effect causes the coxa to swing backwards and the joint comes to a dead stop.

The movements of *T. vandekampi* from walking position to thanatosis and reverse follow a defined sequence (Figure 3A). Some movements of the head, thorax and the appendices may partly happen simultaneously, but there are some benchmarks (Note S1) that must be passed by one component before another component can proceed for mechanical reasons. If this sequence of motions is violated, the weevil is unable to attain a perfect thanatosis position. The functional morphology is designed in a way to maintain the thanatosis position by the interaction of multiple body parts which mechanically block an unwanted opening of appendages. The following sequence of movements and mechanisms is hypothesized based on our animated model and on extensive field observations of the defence behaviour of cryptorhynchine weevils:

1. The tarsi are lifted and nestled backwards along the posterior face of the tibial apices which causes the weevil to lose its hold and fall to the side. The bent tibiae fit into the ventrally sulcate femora, their ventral edge overlapped by the anteroventral ridge of the femora. Thus, each leg forms a compact unit with potential stress relieved from the tibia-femoral joint.
2. The compact femur-tibia-units are pressed together with the left and right legs touching medially. Almost no interspaces are visible between the legs in lateral aspect. Now the tibiae cannot be unfolded since their movement is blocked: the protibiae are blocking each other mesially; the mesotibiae are blocked by the overlapping profemora, and the metatibiae are blocked by the overlapping mesofemora. To allow unfolding of tibiae and tarsi, the pro- and mesocoxae have to be rotated outwards by approx. 10°.
3. The prothoracic acetabula are mesially bordering a comparatively narrow thoracic canal; the procoxae are somewhat D-shaped in cross-section, with their flattened inner faces forming portions of the thoracic canal's lateral wall (Figure 3B). As the rostrum fits tightly into the thoracic canal and the movement of the procoxa is confined to rotation, the latter is mechanically inhibited by the retracted rostrum. Head and prothorax now form one functional unit.
4. The head/prothorax-complex is retracted. Now, the ventral rim of the mesothoracic receptacle overlaps the retracted rostrum's tip ventrally. Thus, the head capsule must be moved forward ca. 57 µm along the beetle's body axis to allow an outward rotation of the procoxae. During thanatosis the antennae are almost fully concealed in the thoracic canal with the rostrum completely covering the opening of the thoracic canal.

Because outward rotation of the trochanteral screw joints is combined with a translatory movement (approx. 0.19 µm/°), the rotation of the protrochanters – and profemora, respectively – is inhibited by the midlegs while the prothorax is pressed against the mesothorax. The posterior surface of the profemur is concave at middle but swollen at the base. This swelling fits tightly into the concave anterior face of the mesocoxa thus blocking the rotation of the latter. The dorsal edge of the mesofemur basally forms an angulation which is posteriorly blocked by the intercoxal ridge of the metathorax tightly opposing it. Since the rotation axis of the mesotrochanter (translation: 0.19 µm/°) is almost perpendicular to the body axis, any elevating rotation is effectively blocked. Such a rotation, which is necessary to bring the leg into walking position, is only possible if the mesocoxa is turned to the side. The metacoxa differs markedly from pro- and mesocoxa as it tilts around two pivotal points. When the metatrochanter is rotating inwards and approaching its resting position (Figures 3D–F), the metafemur is pressed against the intercoxal ridge of the metathorax (Figure 3E). The leverage created causes the metacoxa to swing backwards (Figure 3F) and the metacoxa-trochanteral joint comes to a dead stop. In this position, coxa, trochanter and femur form a functional unit. The metafemur is maximally approximated to the body by the translation of the coxa-trochanteral screw joint, which is largest in the hind leg (0.24 µm/°).

## Discussion

In recent years, complex morphological 3D models based on segmented datasets have been published as PDF files [12], [22], [24] which allow the user to handle and examine relevant structures interactively. Other 3D models containing motion information were published as animated movies but without the option of user-interactivity other than stop-and-go. In fact, PDF files offer the opportunity to combine both motion information and interactivity. Furthermore, their file-size is only a fraction of files published in movie formats e.g. in MOV or MP4.

The recording of 3D data by e.g. CT for studies of functional morphology is ideally coupled with direct movierecording of motion, both taken simultaneously in the best case [7]–[10]. However, such an ideal setting is not always possible: the organisms of interest may be long extinct, too rare, or too shy to observe in a laboratory setting. High-resolution μCT recording suitable for *in vivo* imaging of small-sized specimens still pose radiation doses killing most insects within a few seconds [25]. Obviously, there remains a wide field of conditions where simultaneous recording of both motion and 3D data is impossible.

In many arthropod joints, movements are restricted by the morphology of the corresponding rigid parts, leaving very little play due to precise form closure of the components [26]. Simulations can be performed by interactively moving one component towards its counterpart until the joint reaches one endpoint. Some joints may involve uncertainty where exactly this endpoint is located, but in the described case where the limbs always reach a clearly defined and stable terminal position this was not an issue. The lack of information on the precise timing of motions may be a more serious drawback, especially when it concerns the simulation of complex and highly coordinated movements, such as the movement of two pairs of wings during flight [27], [28] or six pairs of legs performing a running motion [29], [30]. However, while the study of coordinative motion is out of reach without real motion data, it is still possible to investigate the qualitative movement of an isolated limb.

Although these limitations may appear quite restrictive, in the case of *Trigonopterus* weevils the attempt used in the present study proved to be highly effective for understanding the mechanisms of the weevils' defensive morphology (Note S1). The beetle's head and legs interlock mutually and with specific features of thorax and the first abdominal ventrite, presumably to increase its mechanical stability in thanatosis. The protective posture is maintained by minimal muscle activity, and largely by the mechanical interaction of exoskeletal parts. The deterministic interaction of accurately fitting body parts follows a defined sequence, which resembles a piece of engineering and in fact a closer analysis could be of interest to the field of biomimetics. Most aspects of the complex mechanisms could be illustrated in a single PDF 3D model of relatively small data size. While being completely interactive, predefined views illustrate the different mechanisms described above. This underlines the potential of animated 3D models: preserved or extinct species can be brought to life again, at least in the digital world.

## Supporting Information

Figure S1 **Interactive animated 3D reconstruction of the metacoxal joint of** ***Trigonopterus oblongus*** **.** Click on the figure to start interactive 3D view; switch between views by using the menu (Adobe Reader 8.1 or higher required).
(PDF)

Figure S2 **Interactive animated 3D reconstruction of** ***Trigonopterus vandekampi*** **simulating the movements from walking position to thanatosis posture.** Default views illustrating the blocking mechanisms are provided. Click on the figure to start interactive 3D view; switch between views by using the menu (Adobe Reader 8.1 or higher required).
(PDF)

Figure S3 **Optimization of polygon meshes, exemplified with the metacoxa of** ***Trigonopterus oblongus*** **, showing surface (top) and corresponding mesh (bottom).** By a consecutive series of polygon reduction and smoothing, the polygon count – and thus the file size – was reduced to ca. 1/1,000 of its original value without compromising the surface structure while simultaneously reducing labelling artefacts.
(TIF)

Table S1 **List of separate polygon meshes created from labeled exoskeleton parts of** ***Trigonopterus vandekampi*** **.**
(DOCX)

Table S2 **List of the 50 individual articulations animated to create the moving interactive model of** ***Trigonopterus vandekampi*** **.** Note that femora and trochanters do not share movable articulations in the species. Joints between tarsomeres 3 and the minute tarsomeres 4 were neglected.
(DOCX)

Note S1 **Benchmarks of thanatosis cascade.**
(DOCX)

Note S2 **Software changes.**
(DOCX)
