# Supplementary material for: Three-Dimensional Reconstructions Come to Life – Interactive 3D PDF Animations in Functional Morphology
Source: PLoS One. 2014 Jul 16;9(7):e102355. doi: 10.1371/journal.pone.0102355 (PMC4100761; doi:10.1371/journal.pone.0102355)
Supplement: Figure S1 — Interactive animated 3D reconstruction of the metacoxal joint of Trigonopterus oblongus . Click on the figure to start interactive 3D view; switch between views by using the menu (Adobe Reader 8.1 or higher required). (PDF) [file pone.0102355.s001.pdf]

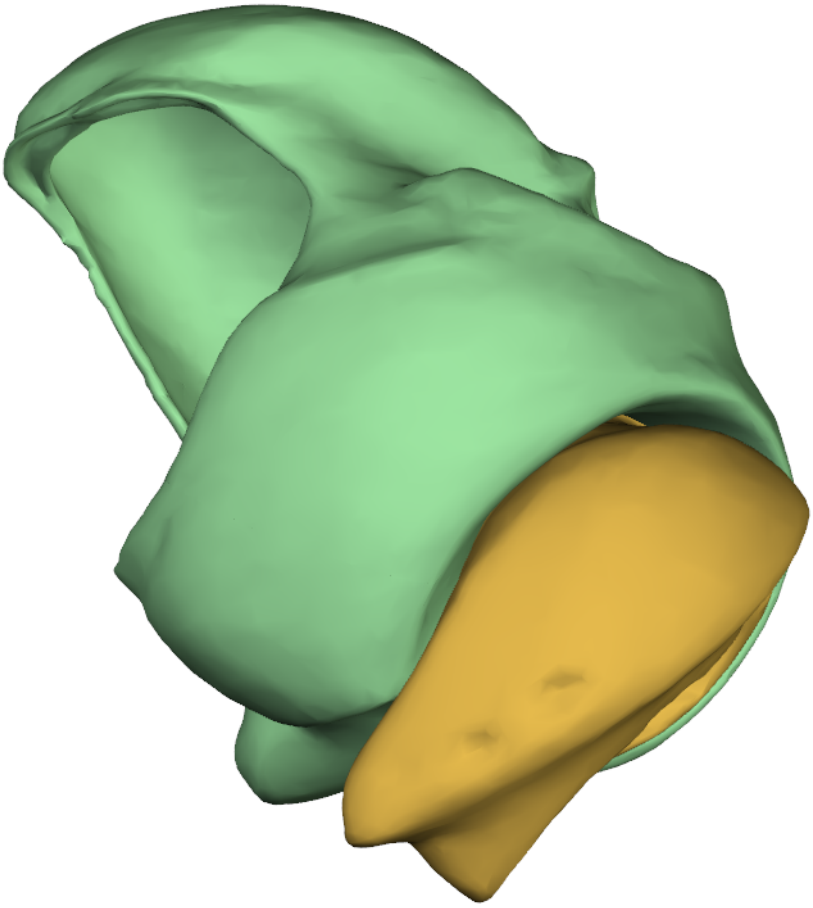

Figure S3. Interactive animated 3D reconstruction of the metacoxal joint of *Trigonopterus oblongus*. Click on the figure to start interactive 3D view; switch between views by using the menu (Adobe Reader 8.1 or higher required).
