# Supplementary material for: Three-Dimensional Reconstructions Come to Life – Interactive 3D PDF Animations in Functional Morphology
Source: PLoS One. 2014 Jul 16;9(7):e102355. doi: 10.1371/journal.pone.0102355 (PMC4100761; doi:10.1371/journal.pone.0102355)
Supplement: Figure S2 — Interactive animated 3D reconstruction of Trigonopterus vandekampi simulating the movements from walking position to thanatosis posture. Default views illustrating the blocking mechanisms are provided. Click on the figure to start interactive 3D view; switch between views by using the menu (Adobe Reader 8.1 or higher required). (PDF) [file pone.0102355.s002.pdf]

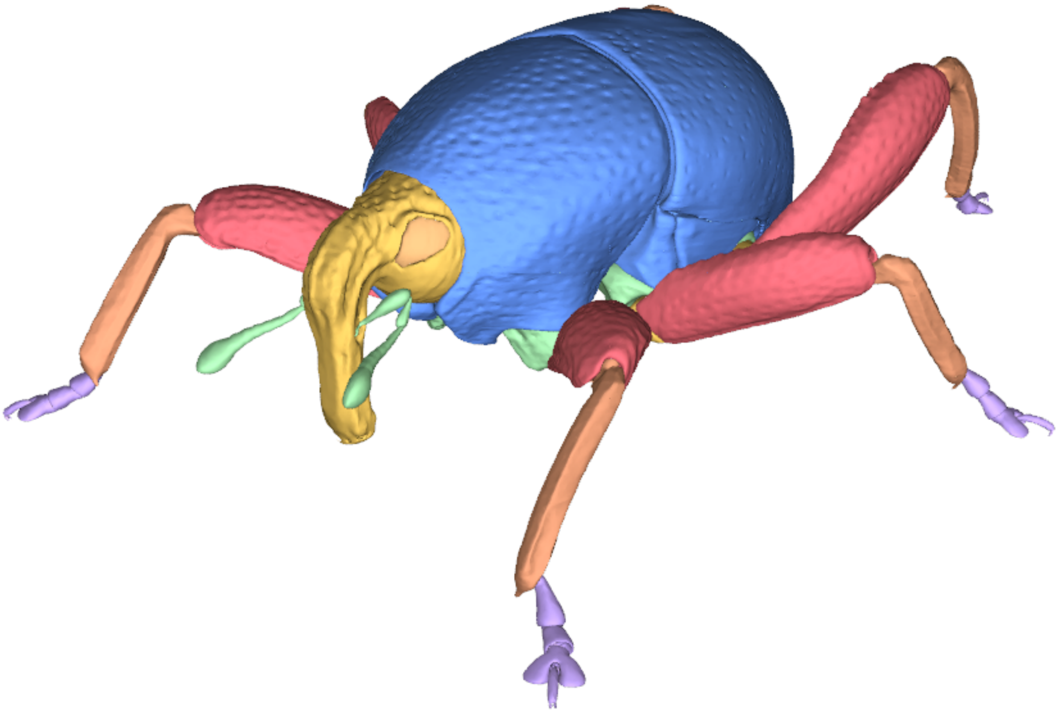

Figure S4. Interactive animated 3D reconstruction of *Trigonopterus vandekampi* simulating the movements from walking position to thanatosis posture. Default views illustrating the blocking mechanisms are provided. Click on the figure to start interactive 3D view; switch between views by using the menu (Adobe Reader 8.1 or higher required).
