# Supplementary figures and images for: Three-Dimensional Reconstructions Come to Life – Interactive 3D PDF Animations in Functional Morphology
Source: PLoS One. 2014 Jul 16;9(7):e102355. doi: 10.1371/journal.pone.0102355 (PMC4100761; doi:10.1371/journal.pone.0102355)

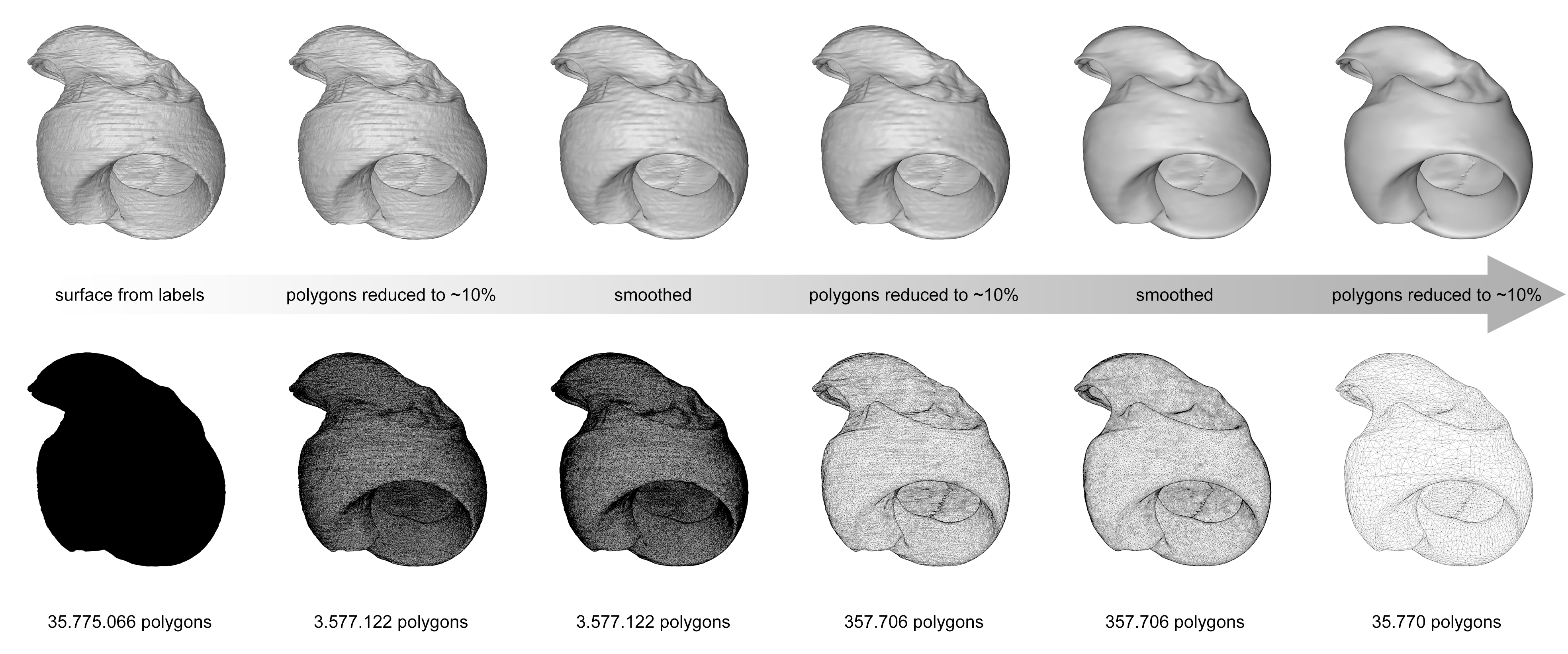

Supplement: Figure S3 — Optimization of polygon meshes, exemplified with the metacoxa of Trigonopterus oblongus , showing surface (top) and corresponding mesh (bottom). By a consecutive series of polygon reduction and smoothing, the polygon count – and thus the file size – was reduced to ca. 1/1,000 of its original value without compromising the surface structure while simultaneously reducing labelling artefacts. (TIF) [file pone.0102355.s003.tif]
