# Supplementary material for: Three-Dimensional Reconstructions Come to Life – Interactive 3D PDF Animations in Functional Morphology
Source: PLoS One. 2014 Jul 16;9(7):e102355. doi: 10.1371/journal.pone.0102355 (PMC4100761; doi:10.1371/journal.pone.0102355)
Supplement: Table S1 — List of separate polygon meshes created from labeled exoskeleton parts of Trigonopterus vandekampi . (DOCX) [file pone.0102355.s004.docx]

| **animated articulations** |  | **joint type** | **QTY** |
| --- | --- | --- | --- |
| prothorax-head capsule |  | form & force guided | 1 |
| head capsule-scape |  | ball-and-socket | 2 |
| scape-pedicel |  | hinge | 2 |
| pedicel-flagellum |  | ball-and-socket | 2 |
| prothorax-mesothorax  prothorax-procoxa |  | form & force guided  rotative | 1  2 |
| procoxa-protrochanter |  | screw-and-nut | 2 |
| profemur-protibia |  | hinge | 2 |
| protibia-protarsus 1 |  | ball-and-socket | 2 |
| protarsomere 1-protarsomere 2 |  | freely movable | 2 |
| Protarsomere 2-protarsomere 3 |  | freely movable | 2 |
| protarsomere 4-propretarsus  mesothorax-mesocoxa |  | freely movable  rotative | 2  2 |
| mesocoxa-mesotrochanter |  | screw-and-nut | 2 |
| mesofemur-mesotibia |  | hinge joint | 2 |
| mesotibia-mesotarsus 1 |  | ball-and-socket | 2 |
| mesottarsomere 1-mesotarsomere 2 |  | freely movable | 2 |
| mesotarsomere 2-mesotarsomere 3 |  | freely movable | 2 |
| mesotarsomere 4-mesopretarsus |  | freely movable | 2 |
| metathorax-metacoxa |  | dicondylic | 2 |
| metacoxa-metatrochanter |  | screw-and-nut | 2 |
| metafemur-metatibia |  | hinge | 2 |
| metatibia-metatarsomere 1 |  | ball-and-socket | 2 |
| metatarsomere 1-metatarsomere 2  metatarsomere 2-metatarsomere 3 |  | freely movable  freely movable | 2  2 |
| metatarsomere 4-metapretarsus |  | freely movable | 2 |

**Table S1.** List of separate polygon meshes created from labeled exoskeleton parts of *Trigonopterus vandekampi*.
