# Supplementary material for: Three-Dimensional Reconstructions Come to Life – Interactive 3D PDF Animations in Functional Morphology
Source: PLoS One. 2014 Jul 16;9(7):e102355. doi: 10.1371/journal.pone.0102355 (PMC4100761; doi:10.1371/journal.pone.0102355)
Supplement: Table S2 — List of the 50 individual articulations animated to create the moving interactive model of Trigonopterus vandekampi . Note that femora and trochanters do not share movable articulations in the species. Joints between tarsomeres 3 and the minute tarsomeres 4 were neglected. (DOCX) [file pone.0102355.s005.docx]

| **structure** |  | **component** |
| --- | --- | --- |
| head |  | head capsule |
|  |  | compound eyes |
|  |  | scape |
|  |  | pedicel |
|  |  | flagella |
| prothorax |  | prothoracic sclerites |
|  |  | cryptopleuron |
|  |  | procoxa |
|  |  | protrochanter |
|  |  | profemur |
|  |  | protibia |
|  |  | protarsomere 1 |
|  |  | protarsomere 2 |
|  |  | protarsomere 3 |
|  |  | protarsomere 4 |
|  |  | propraetarsus |
|  |  | propraetarsal claws |
| mesothorax |  | mesothoracic sclerites |
|  |  | elytra (as single object) |
|  |  | mesocoxa |
|  |  | mesotrochanter |
|  |  | mesofemur |
|  |  | mesotibia |
|  |  | mesotarsomere 1 |
|  |  | mesotarsomere 2 |
|  |  | mesotarsomere 3 |
|  |  | mesotarsomere 4 |
|  |  | mesopraetarsus |
|  |  | mesopraetarsal claws |
| metathorax |  | metathoracic sclerites |
|  |  | metacoxa |
|  |  | metatrochanter |
|  |  | metafemur |
|  |  | metatibia |
|  |  | metatarsomere 1 |
|  |  | metatarsomere 2 |
|  |  | metatarsomere 3 |
|  |  | metatarsomere 4 |
|  |  | metapraetarsus |
|  |  | metapraetarsal claws |
| abdomen |  | abdominal sclerites 1 & 2 |
|  |  | abdominal sclerite 3 |
|  |  | abdominal sclerite 4 |
|  |  | abdominal sclerite 5 |

**Table S2.** List of the 50 individual articulations animated to create the moving interactive model of *Trigonopterus vandekampi*. Note that femora and trochanters do not share movable articulations in the species. Joints between tarsomeres 3 and the minute tarsomeres 4 were neglected.
