# Supplementary material for: Three-Dimensional Reconstructions Come to Life – Interactive 3D PDF Animations in Functional Morphology
Source: PLoS One. 2014 Jul 16;9(7):e102355. doi: 10.1371/journal.pone.0102355 (PMC4100761; doi:10.1371/journal.pone.0102355)
Supplement: Note S1 — Benchmarks of thanatosis cascade. (DOCX) [file pone.0102355.s006.docx]

**Note S1.** Benchmarks of thanatosis cascade

- the head must be tilted less than 6° dorsad from its ventral terminal position, so the rostrum gets pushed into the thoracic receptacle during the retraction of the prothorax. The shape of the receptacle would ensure that the rostrum is passively placed into the 0° terminal position.
- the antennae must be folded to the ventral face of the rostrum while the head is ca. 20° dorsad from its ventral terminal position, to avoid them being trapped between the rostrum and the edge of the prothoracic canal.
- the rotations of the head and the procoxae should be completed at the same time. The rostrum only fits into the thoracic canal when the procoxae are rotated less then ca. 8° from their terminal position.
- the femora must be at least 34° from the thanatosis position to allow the fully extended tibiae being folded.
- the profemur must be in its final position before the retraction of the prothorax, otherwise its base collides with the mesocoxa. The final 10° rotation of the procoxa could be achieved passively by pressure from the mesothorax.
- the midlegs must be in their final position before the retraction of the prothorax. The final 8° rotation of the mesocoxa could be achieved passively by pressure from the profemur.
- the folding of the metatibia should be completed before or simultaneous with the folding of the midlegs to avoid collision with the mesofemora.
