# Supplementary material for: Three-Dimensional Reconstructions Come to Life – Interactive 3D PDF Animations in Functional Morphology
Source: PLoS One. 2014 Jul 16;9(7):e102355. doi: 10.1371/journal.pone.0102355 (PMC4100761; doi:10.1371/journal.pone.0102355)
Supplement: Note S2 — Software changes. (DOCX) [file pone.0102355.s007.docx]

**Note S2.** Software changes

*Right Hemisphere® Deep Exploration*

In 2011, Right Hemisphere® was acquired by SAP Deutschland AG & Co. KG. The software package Deep Exploration that was employed in our study, is now called SAP Visual Enterprise Author.

*Adobe® Acrobat®*

Embedding interactive 3D models into PDF documents was first introduced by Adobe in 2005 with the release of Acrobat 3D 7.0. In Version 9.0 (2008), 3D support was now part of Acrobat Pro Extended (Windows only), which we used in our study. With the arrival of Acrobat X (2010), Adobe suspended the creation of 3D PDF documents and outsourced 3D support to the company Tetra 4D, which now provides the 3D PDF converter plugins for Acrobat necessary to create 3D PDF documents.
